# Risk Factors Associated With Echinococcosis in the General Chinese Population: A Meta-Analysis and Systematic Review

**DOI:** 10.3389/fpubh.2022.821265

**Published:** 2022-05-17

**Authors:** Tiantian Zhang, Bin Li, Yuying Liu, Shou Liu

**Affiliations:** Department of Public Health, Medical College, Qinghai University, Xining, China

**Keywords:** risk factor, human, meta-analysis, echinococcosis, China

## Abstract

**Background:**

Echinococcosis is a severe zoonotic disease that imposes a substantial burden on human life. This meta-analysis aimed to summarize available data on the prevalence of human echinococcosis and identify the key risk factors for echinococcosis in the Chinese general population.

**Methods:**

Relevant studies were comprehensively searched in the PubMed, EMBASE, Web of Science, Cochrane, Chinese National Knowledge Infrastructure (CNKI), Chongqing VIP Information (VIP), Wanfang and SinoMed databases until August 22, 2020. A random-effects model was used to estimate the pooled odds ratio (OR) and 95% confidence interval (95% CI). The I^2^ and Q statistics were calculated to evaluate the heterogeneity, and potential sources of heterogeneity were identified using sensitivity analysis and subgroup analysis. Publication bias was estimated by funnel plots and Egger's test.

**Results:**

A total of 1026 studies were identified through the database search, of which 26 were eligible for this meta-analysis. The pooled prevalence of AE and CE were 2.88% and 5.66%, respectively. Ethnicity (OR = 2.93, 95% CI: 1.81–4.75; I^2^ = 0), herdsman occupation (OR = 2.66, 95% CI: 2.25–3.14; I^2^ = 8.0%), not washing hands before meals (OR = 2.40, 95% CI: 1.34–4.28; I^2^ = 82.8%) and being female (OR = 1.45, 95% CI: 1.26–1.66; I^2^ = 33.9%) were risk factors for AE. The top five risk factors for CE were ethnicity (OR = 3.18, 95% CI: 1.55–6.52; I^2^ = 79.2%), nomadism (OR = 2.71, 95% CI: 1.65–4.47; I^2^ = 55.8%), drinking nonboiled water (OR = 2.47, 95% CI: 1.36–4.47; I^2^ = 85.7), feeding viscera to dogs (OR = 2.35, 95% CI: 1.89–2.91; I^2^ = 21.5%), and herdsman occupation (OR = 2.19, 95% CI: 1.67–2.86; I^2^ = 85.1%).

**Conclusions:**

This study generalized articles that have contributed to our current understanding of the epidemic of human echinococcosis (AE and CE) in China over the years. The results support that the ethnicity and dog-related factors are major risk factors for both CE and AE. The identification of echinococcosis risk factors may aid researchers and policymakers in improving surveillance and preventive measures aimed at reducing *Echinococcus granulosus* and *Echinococcus multilocularis* infection in humans.

## Introduction

Echinococcosis is widely known as a zoonotic and natural-focal disease in which HUMANs play the role of aberrant, dead-end intermediate hosts. Cystic echinococcosis (CE) and alveolar echinococcosis (AE) are the two most common forms of human echinococcosis and are caused by the larval stages of *Echinococcus granulosus* and *Echinococcus multilocularis*, respectively (1). Dogs are the usual definitive host of *E. granulosus* (2), whereas dogs and foxes are the main definitive hosts of *E. multilocularis* (3). Both are transmitted by the fecal-oral route through contact with infected definitive hosts or with food or water contaminated with *E. granulosus* or *E. multilocularis* eggs. The annual numbers of new cases of CE and AE are estimated at 188,000 and 18,200, respectively, leading to a corresponding total of 184,000 and 666,000 disability-adjusted life years (DALYs) (4). AE has a higher mortality rate than that of CE, which is one of the major reasons for the greater global AE burden (5); It is also called “worm cancer” (6).

Echinococcus parasites can inhabit any part of the human body, but mainly favor the liver, lungs, brain and abdomen. Once a parasite attaches to the human body, health deteriorates. CE is endemic in pastoral areas around the world, where it is often maintained by herders feeding viscera from infected ruminants to dogs. For AE, in addition to the original life cycle in wild canids, a life cycle has also been established in domestic dogs, which are the most significant transmitters of AE in China (7). AE infection is maintained through dog predation on small rodents (8). Therefore, compared to those of *E. granulosus*, the potential risk factors for *E. multilocularis* are more complex because its life cycle involves multiple wild canids as final hosts and a large number of small mammals (mostly rodents) as intermediate hosts (9).

To date, many studies have examined the risk factors for echinococcosis, each study focusing on different areas. The geographic distribution and prevalence of echinococcosis vary from region to region and are mainly influenced by biological and abiotic factors. The biological factors include host species, transmission mechanism, density and prevalence among definitive hosts (5), and the abiotic factors include environmental, socioeconomic and behavioral factors. A previous study (10) on environmental and socioeconomic risk factors for CE in western China showed that the ratio of grassland positively correlated with the prevalence of human CE, whereas the gross domestic product and land surface temperature (in spring) were independently negatively correlated with disease prevalence. Wang Qian (11) reported that owning fox hides, letting flies land on food, using open streams as drinking water sources and playing with dogs were significant behavioral risk factors for AE. However, it is difficult to identify the primary high-risk factors for echinococcosis because of differences in the groups, type of echinococcosis and study region among studies. Therefore, the present meta-analysis pooled the results of previous studies and aimed to analyze the main risk factors for AE and CE.

## Materials and Methods

### Search Strategy

We followed the Preferred Reporting Items for Systematic Reviews and Meta-analyses (PRISMA) guidelines when performing the literature search. Two researchers (T.Z. and B.L.) independently searched for relevant articles published in four English (PubMed, Embase, Web of Science, and the Cochrane Library) and four Chinese (China National Knowledge Infrastructure, China Science and Technology Journal Database, Wanfang Data and SinoMed) databases from their inception to August 22, 2020. The search terms were [(“echinococcosis” OR “echinococcoses” OR “echinococcus infection” OR “hydatidosis” OR “hydatidoses” OR “hydatid cyst” OR “hydatid disease” OR “echinococcus granulosus infection”) AND (“risk factor” OR “population at risk” OR “homo sapiens” OR “man” OR “human”) AND (“People's Republic of China” OR “Chinese” OR “China”)]. In addition, the references of reviews and meta-analyses were manually screened to identify additional potentially relevant studies.

### Eligibility Criteria

Studies were eligible if they met the following inclusion criteria: (1) the research was conducted with Chinese residents; (2) the diagnoses of AE and CE were based on a combination of serological and ultrasonic methods; and (3) the odds ratios (ORs) and their 95% confidence intervals (95% CIs) could be obtained directly or calculated from the study.

Studies were excluded if (1) the publications were neither in Chinese nor in English; (2) the sample size was ≤ 30 (12); (3) no risk factors were reported; (4) several articles were based on data from one study sample, only the article with the most comprehensive results was included; and (5) the publication was low quality based on its overall Newcastle-Ottawa Scale (NOS) or Agency for Healthcare Research and Quality (AHRQ) score.

### Quality Assessment

Two authors (TZ and BL) independently assessed the quality of the studies. We employed NOS and AHRQ scores to assess the quality of cross-sectional studies and case-control studies. NOS scores range from 0 to 8; scores of 7–8, 4–6 and 0–3 indicate a study of high, medium and low quality, respectively (13, 14). AHRQ scores are between 0 and 11, with scores of 8–11 indicating high quality and scores of 4–7 and 0–3 indicating moderate and low quality, respectively (15). Any disagreements during this process were resolved by discussion with the third author YL.

### Data Extraction

Two authors (TZ and BL) independently extracted data and information from the studies including the first author, year of publication, region, type of echinococcosis (AE or CE), study design, sample size, number of positive cases, participant age in years, sex, participant race/ethnicity, whether the participants were herdsmen, ang/or raised dogs, the kind/number of animal hosts, participant hand washing status, and the OR value and its 95% CI, or the original data from which the OR could be calculated.

### Meta-Analysis

The ORs and 95% CIs of the associated factors were pooled using random-effects models if at least three studies reported data on the same factor (16). The results were represented using forest plots. Additionally, the Q test was used to test the level of heterogeneity between studies; the percentage of total variation in the results due to heterogeneity was assessed based on the I^2^ statistic. An I^2^ < 25%, 25–50%, 50–75% and 75–100% represents no, moderate, large and extreme heterogeneity, respectively (17). In this study, *P* < 0.05 and I^2^ > 50% were considered to indicate significant heterogeneity between studies (18). Sensitivity analysis using the leave-one-out method was performed to evaluate the stability and reliability of the results. In addition, Egger's test (19) and funnel plots were used to test for the presence of publication bias. The prevalence of AE and CE in endemic areas was estimated using a random-effects model that combined the prevalence reported in previous cross-sectional studies.

We employed subgroup analysis to explore the source of heterogeneity on the basis of study design (i.e., case-control study or cross-sectional study) and geographic distribution of the studies (Ningxia, Qinghai or Xinjiang). Data were analyzed using the R (4.0.0) package meta (20, 21), and *p* < 0.05 was considered statistically significant.

## Results

### Study Selection

**Figure 1:**
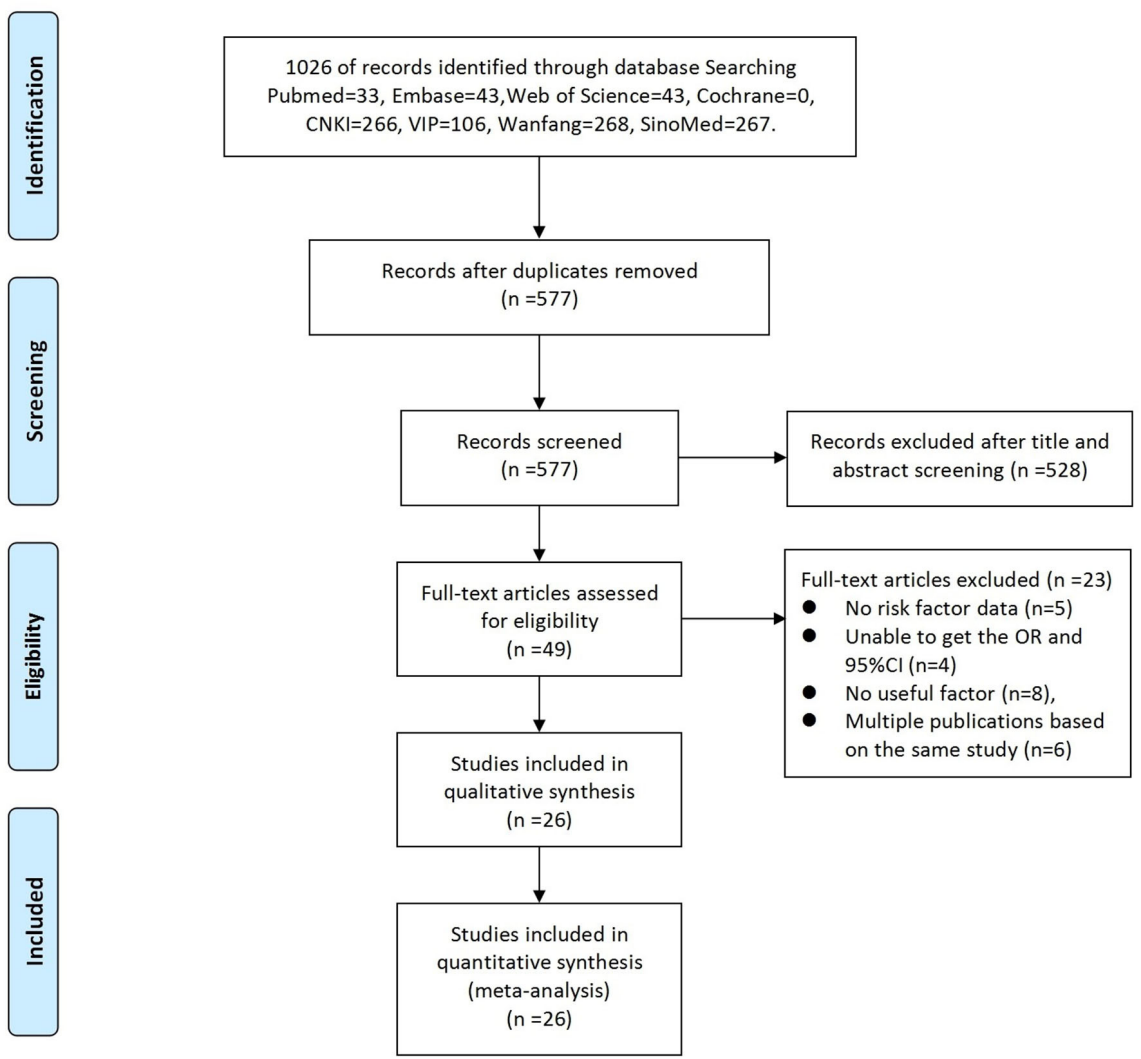
Summary of the literature search and study selection.

A total of 1,026 articles were originally identified; of these, 449 were excluded as duplicates. Thus, 577 studies were screened; of these, 26 (Table 1) were eligible and subsequently included in this meta-analysis. The literature selection process is detailed in Figure 1, and the basic characteristics of the included studies are shown in Table 1. All the cross-sectional studies (20/26) were of medium quality. The case-control studies (6/26) included three medium-quality and three high-quality studies (Table 1). No cohort studies were included in our analysis.

**Table 1:** Main characteristics of the included studies.

**No**.	**References**	**Year of publication**	**Region**	**Type of echinococcosis**	**Study design**	**Sample size**	**Positive case**	Risk factors^*^	**Quality score^#^**
1	Zeng et al. (22)	2020	Western China	CE	Cross-sectional	470,400	32,928	11	4
2	He et al. (23)	2019	Sichuan	CE	Case-control	-	-	1,11,12	8
3	Li et al. (24)	2019	Tibet	CE	Cross-sectional	80,384	1,371	1,7	6
4	Wu et al. (25)	2018	Tibetan plateau	CE	Case-control	378	189	3,5,8,10,11	7
5	Li et al. (26)	2017	Qinghai	CE	Cross-sectional	600	11	1	4
6	Yuan et al. (27)	2017	Western China	CE	Cross-sectional	5,813	90	5,8,10,9,13	4
7	He et al. (28)	2017	Yunnan	AE	Cross-sectional	9,460	348	1	5
				CE				1	
8	Li et al. (29)	2015	Gansu	CE	Cross-sectional	972	92	8,12	5
9	Yang et al. (30)	2015	Xinjiang	CE	Cross-sectional	42,356	159	7	5
10	Qi et al. (31)	2015	Xinjiang	CE	Cross-sectional	532	23	1,7	4
11	Luo et al. (32)	2014	Qinghai	CE	Cross-sectional	23,445	1,048	1,2,7	6
12	Giraudoux et al. (33)	2013	Tibetan plateau	AE	Cross-sectional	15,614	577	1,2,7	5
13	Bai et al. (34)	2013	Xinjiang	CE	Cross-sectional	869	11	1	4
14	Wang et al. (35)	2009	Xinjiang	CE	Case-control	5,037	141	2,3,7	6
15	Yuan et al. (36)	2011	Gansu	CE	Case-control	75	25	3,8	6
16	Feng et al. (37)	2011	Ningxia	AE	Cross-sectional	6,039	89	1	5
				CE				1	
17	Wu et al. (38)	2010	Ningxia	AE	Cross-sectional	3,196	72	1,3,5	5
				CE				1,3,5,8	
18	Zhong et al. (39)	2009	Xinjiang	CE	Cross-sectional	3,691	56	1,7	5
19	Li et al. (40)	2008	Ningxia	CE	Case-control	303	101	3,9,13	7
20	Yang et al. (41)	2008	Ningxia	CE	Case-control	387	129	8,9,13	6
21	Yang et al. (42)	2006	Ningxia	AE	Cross-sectional	4,773	96	1	5
				CE			75	1,9	
22	Wang et al. (11)	2006	Sichuan	AE	Cross-sectional	7,138	223	1,2,3,4,5, 6,7	5
23	Schantz et al. (43)	2003	Qinghai	AE	Cross-sectional	3,703	31	1,2,5,6,7,8,9,10	6
				CE			243	1,2,5,7,8,9,10	
24	Wang et al. (44)	2001	Sichuan	AE	Cross-sectional	1,858	43	1,3,4,5,6	4
				CE			65	1,3,5,10,12	
25	Wu et al. (45)	2001	Qinghai	CE	Cross-sectional	817	38	1,2	4
26	Craig et al. (46)	2000	Gansu	AE	Cross-sectional	2,482	84	3,4	5

* *1. Sex (Female/Male); 2. Ethnicity (Tibetan/Han); 3. Dog ownership; 4. Contact with fox hides; 5. Not washing hands before meals; 6. Playing with dogs; 7. Herdsman occupation; 8. Feeding viscera to dogs; 9. Drinking nonboiled water; 10. Presence of stray dogs; 11. Number of household dog (with each addition); 12. Nomadism; 13. Eating raw vegetables*.

# *We used NOS and AHRQ, respectively, in cross-sectional and case-control study*.

*AE, alveolar echinococcosis; CE, cystic echinococcosis*.

Overall, the included studies covered 690,322 individuals (AE studies = 54,338, CE studies = 635,984), of which 38,358 had echinococcosis (AE cases = 1,588, CE cases = 36,770) according to the combined diagnosis based on ultrasound and serological methods. The included studies varied in location, including the Tibetan Autonomous Region (*n* = 1), Qinghai Province (*n* = 4), Western China (*n* = 2), Yunnan Province (*n* = 1), Gansu Province (*n* = 3), Xinjiang Province (*n* = 5), Ningxia Province (*n* = 5), Sichuan Province (*n* = 3), and the Tibetan Plateau (*n* = 2). Three of the included studies reported only on AE-infected patients, seventeen reported only on CE-infected patients, and six studies reported on both AE-and CE-infected patients.

**Figure 2:**
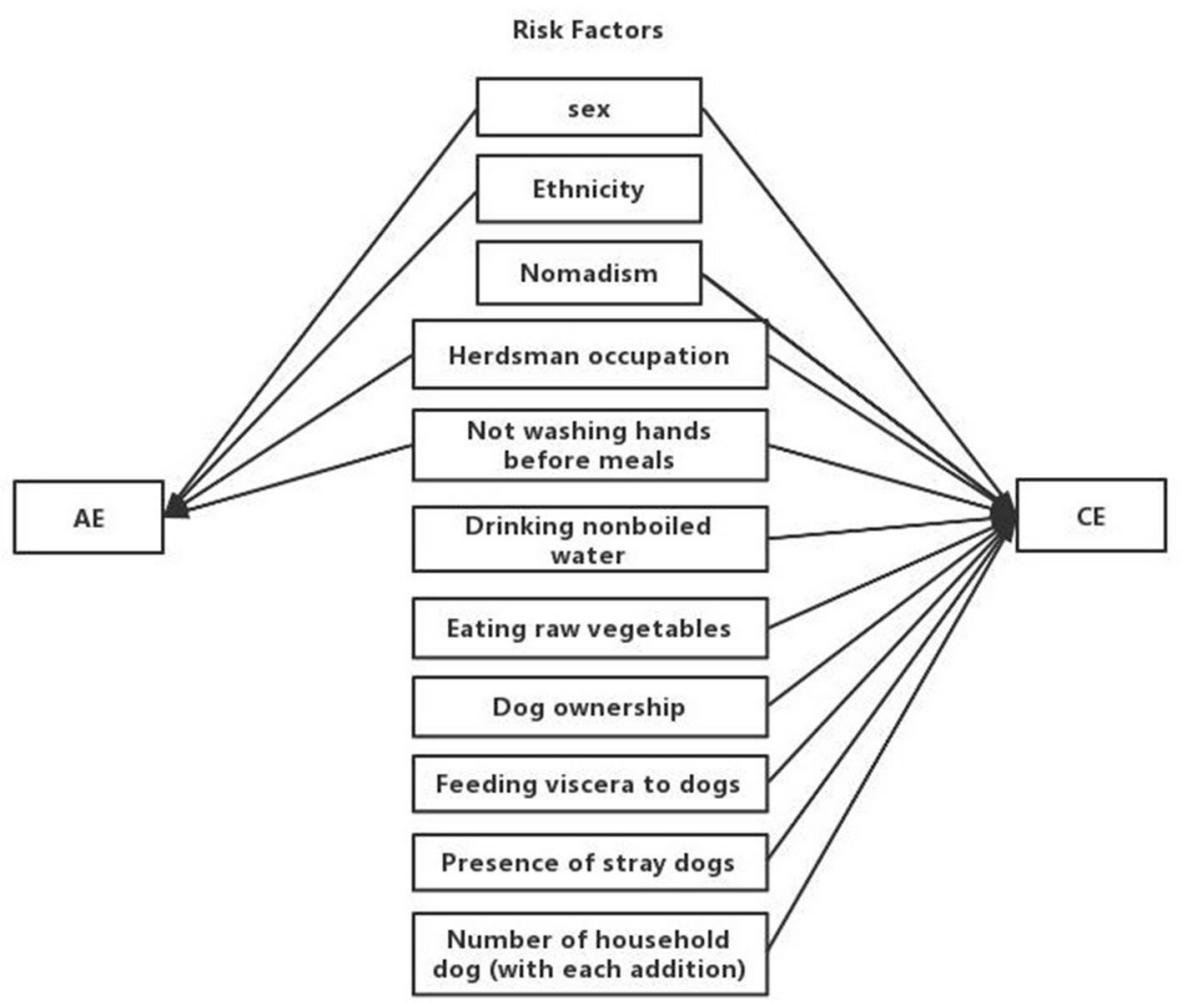
Concept map of risk factors for AE and CE.

In total, thirteen potential risk factors reported were included in the meta-analysis: participant sex, ethnicity, dog ownership, contact with fox hides, not washing hands before meals, playing with dogs, herdsman occupation, feeding viscera to dogs, drinking nonboiled water, nomadism, eating raw vegetables, the presence of stray dogs, and number of household dogs. The risk factors associated with AE and CE are shown in Figure 2.

Twenty cross-sectional studies conducted before August 2020 reported the prevalence of echinococcosis; the pooled prevalence of AE and CE in endemic districts were 2.34% (95% CI: 1.74–3.13%) and 4.45% (95% CI: 2.53–7.71%), respectively.

### Potential Risk Factors for AE

**Figure 3:**
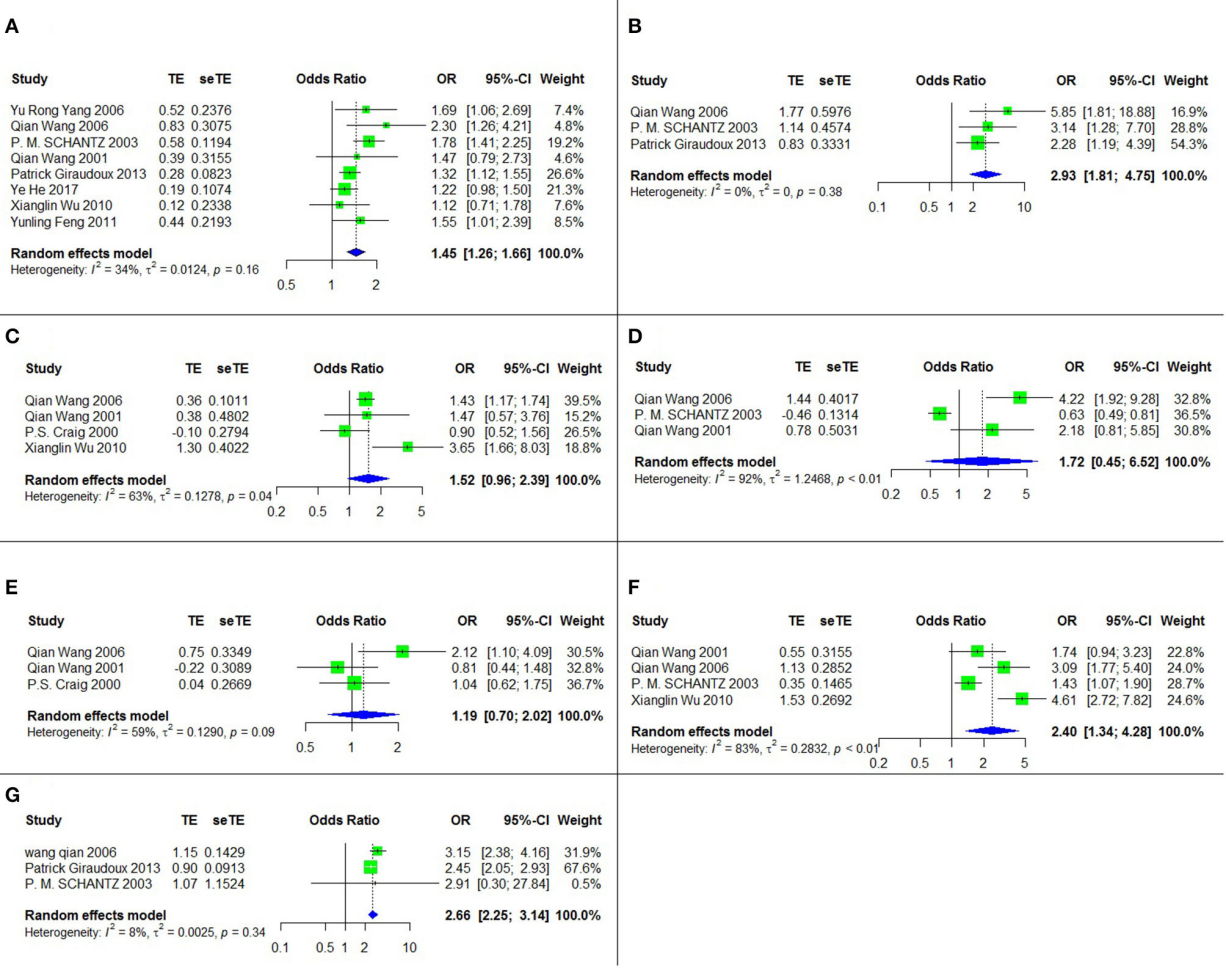
The forest chart of AE factors. **(A)** Sex (female/male); **(B)** Ethnicity (Tibetan/Han); **(C)** Dog ownership; **(D)** Playing with dogs; **(E)** Contanct with fox hides; **(F)** Not washing hands before meals; **(G)** Herdsman occupation.

Seven risk factors for AE were indicated among the studies, and a meta-analysis was executed on nine cross-sectional studies (11, 28, 33, 37, 38, 42–45). The results of the meta-analysis and forest plots are summarized in Table 2, Figure 3.

**Table 2:** Result of echinococcosis risk factors meta-analysis.

**Risk factors**	**Number of studies included**	**Type of echinococcosis**	**Sample size**	**Positive cases**	**Test of heterogeneity**			**OR**	**95% CI**	**Test of overall effect**	
					**Q**	***P***	**I^**2**^ (%)**			**Z**	***P***
Sex (Female/Male)	8	AE	51,781	1,479	10.59	0.158	33.9	1.45	1.26–1.66	6.73	<0.001
Ethnicity (Tibetan/Han)	3	AE	26,455	831	1.92	0.382	0	2.93	1.81–4.75	4.38	<0.001
Dog ownership	4	AE	14,674	422	8.15	0.043	63.2	1.52	0.96–2.39	1.78	0.075
Playing with dogs	3	AE	12,699	297	24.44	<0.001	91.8	1.72	0.45–6.52	0.80	0.424
Contact with fox hides	3	AE	11,478	350	4.82	0.090	58.5	1.19	0.70–2.02	0.64	0.523
Not washing hands before meals	4	AE	15,895	369	17.49	0.001	82.8	2.40	1.34–4.28	2.96	0.003
Herdsman occupation	3	AE	26,455	831	2.17	0.337	8.0	2.66	2.25–3.14	12.63	<0.001
Sex (Female/Male)	14	CE	139,367	3,450	89.4	<0.001	85.5	1.30	1.11–1.53	3.27	0.001
Ethnicity (Tibetan/Han)	4	CE	33,002	1,470	14.44	0.002	79.2	3.18	1.55–6.52	3.16	0.002
Dog ownership	6	CE	10,847	593	9.88	0.079	49.4	1.54	1.09–2.17	2.46	0.014
Not washing hands before meals	5	CE	14,948	659	21.44	<0.001	81.3	2.05	1.35–3.10	3.39	0.001
Herdsman occupation	7	CE	159,148	3,041	40.25	<0.001	85.1	2.19	1.67–2.86	5.71	<0.001
Feeding viscera to dogs	7	CE	14,524	840	7.64	0.266	21.5	2.35	1.89–2.91	7.78	<0.001
Drinking nonboiled water	5	CE	14,979	638	27.91	<0.001	85.7	2.47	1.36–4.47	2.99	0.003
Presence of stray dogs	4	CE	11,752	587	6.11	0.106	50.9	1.75	1.15–2.65	2.64	0.008
Number of household dog (with each addition)	3	CE	470,778	33,117	7.48	0.024	73.2	1.66	1.17–2.34	2.85	0.004
Nomadism	3	CE	2,830	157	4.53	0.104	55.8	2.71	1.65–4.47	3.92	<0.001
Eating raw vegetables	3	CE	6,503	320	0.84	0.658	0	1.86	1.47–2.35	5.16	<0.001

*AE, alveolar echinococcosis; CE, cystic echinococcosis; OR, Odds Ratio; 95% CI, 95% confidence interval*.

Four of these seven risk factors were statistically significant. They are listed according to the strength of the correlation as follows: ethnicity (Tibetan vs. Han) (OR = 2.93, 95% CI: 1.81–4.75; *p* < 0.001), herdsman occupation (OR = 2.66, 95% CI: 2.25-3.14; *p* < 0.001), not washing hands before meals (OR = 2.40, 95% CI: 1.34–4.28; *p* = 0.003) and sex (female vs. male) (OR = 1.45, 95% CI: 1.26–1.66; *p* < 0.001).

### Potential Risk Factors for CE

**Figure 4:**
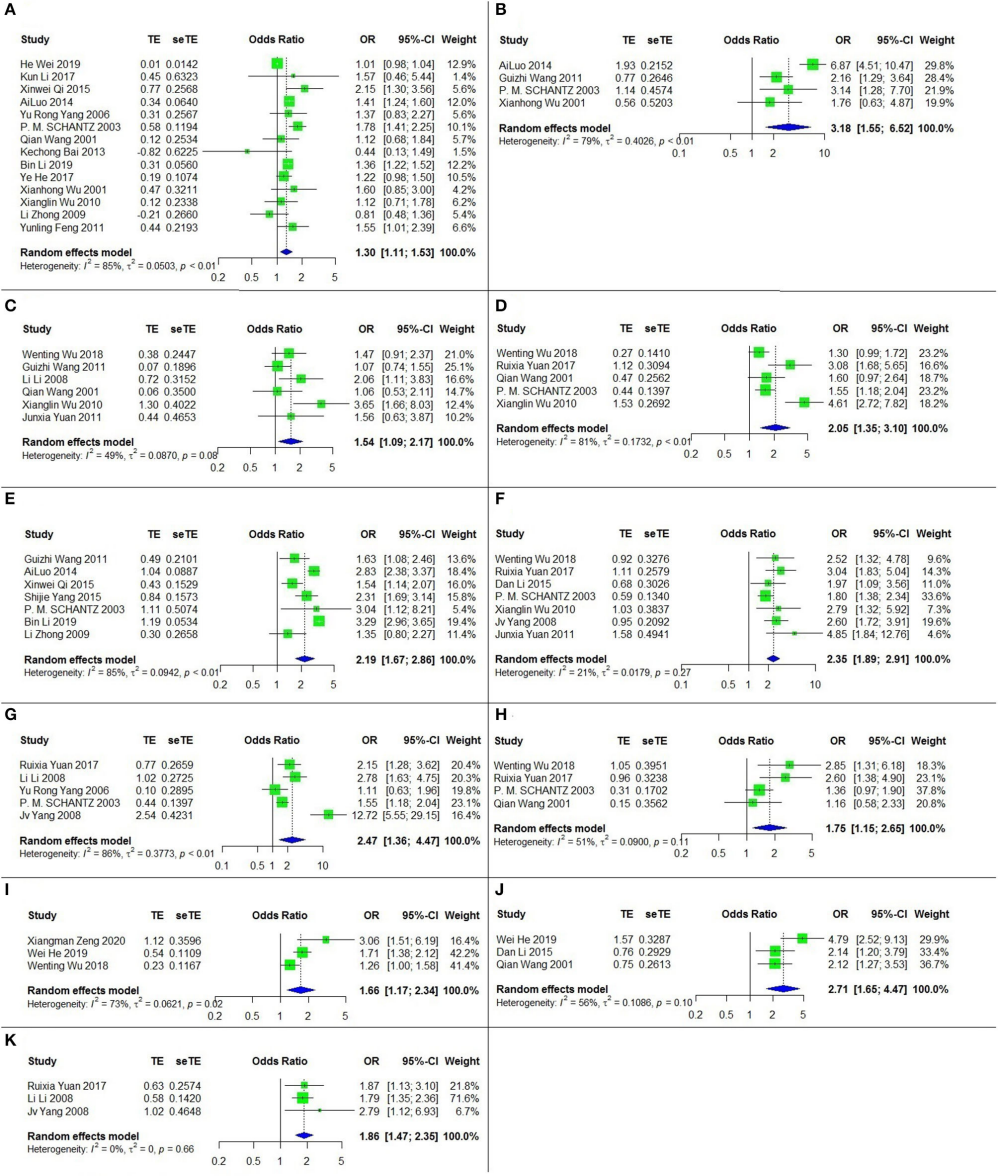
The forest chart of CE factors. **(A)** Sex (female/male); **(B)** Ethnicity (Tibetan/Han); **(C)** Dog ownership; **(D)** Not washing hands before meals; **(E)** Herdsman occupation; **(F)** Feeding viscera to dogs; **(G)** Drinking nonboiled water; **(H)** Presence of stray dogs; **(I)** Number of household dog (with each addition); **(J)** Nomadism; **(K)** Eating raw vegetables.

Eleven risk factors for CE were recognized among the relevant studies, and a meta-analysis was performed on twenty-three papers including six case-control studies and seventeen cross-sectional studies (Table 1). The results are shown in Table 2, Figure 4.

All of these eleven risk factors were statistically significant. The top three, according to the strength of the correlation, were ethnicity (Tibetan vs. Han) (OR = 3.18, 95% CI: 1.55–6.52; *p* = 0.002), nomadism (OR = 2.71, 95% CI: 1.65-4.47; *p* < 0.001) and drinking nonboiled water (OR = 2.47, 95% CI: = 1.36–4.47; *p* = 0.003).

### Sensitivity Analysis

The sensitivity analysis revealed that the results were stable for most of the risk factors. However, when we removed two of the studies [(27, 38)] on AE, the heterogeneity of playing with dogs and dog ownership declined markedly, and their corresponding results became statistically significant. Similarly, when we removed three of the studies related to CE (23, 25, 32), the heterogeneity of sex, ethnicity and the presence of stray dogs dropped below 50%. More details are shown in Supplementary Material.

### Publication Bias

**Figure 5:**
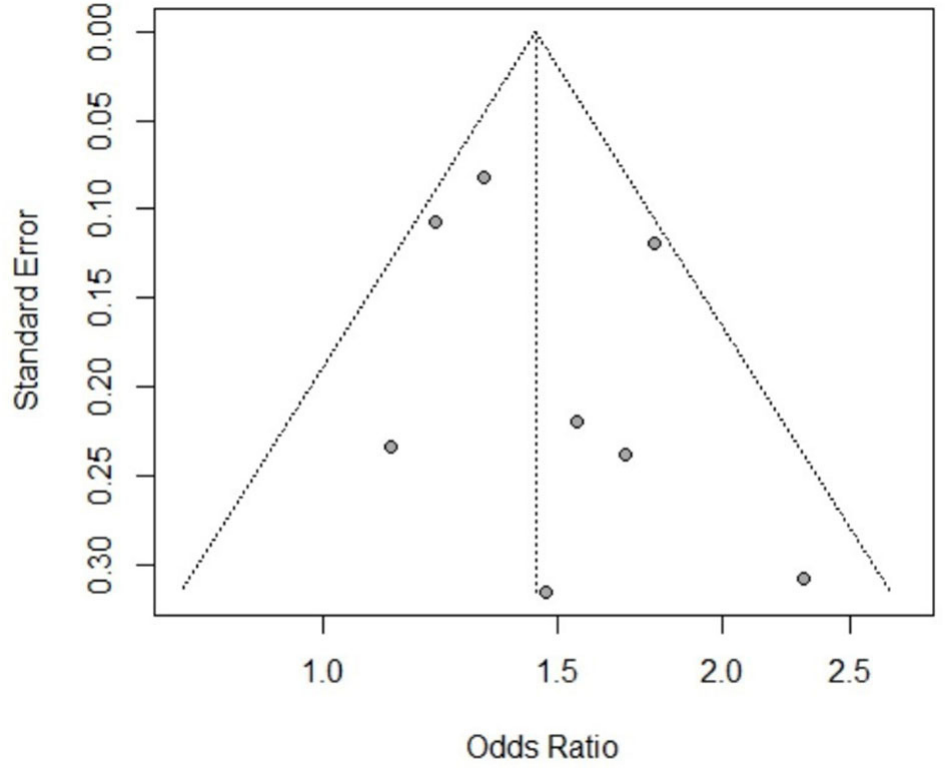
Funnel chart of AE risk factor sex.

The publication bias was assessed for all the risk factors included in this study (see Supplementary Material). Based on the results of Egger's test and the funnel charts, three of the risk factors for CE (participant sex, herdsman occupation and feeding viscera to dogs) exhibited publication bias. Other risk factors did not exhibit bias; for instance, the p value of Egger's test for the participant sex, as a risk factor of AE, was >0.05, and the funnel chart was largely symmetric (Figure 5).

### Subgroup Analysis

The study design-specific subgroup analysis examined only risk factors for CE because all of the AE studies had a cross-sectional design. The results are shown in Supplementary Material. In the case-control studies, two risk factors were significant: dog ownership (OR = 1.35, 95% CI: 1.03-1.83; *p* = 0.029) and feeding viscera to dogs (OR = 2.76, 95% CI: 2.00–3.83; *p* < 0.001). In the cross-sectional studies, seven risk factors were identified. The top three, according to the strength of the correlation, were ethnicity (OR = 3.71, 95% CI: 1.60–8.59; *p* = 0.002), not washing hands before meals (OR = 2.37, 95% CI: 1.40–4.00; *p* = 0.003) and herdsman occupation (OR = 2.30, 95% CI: 1.74–3.04; *p* < 0.001). The heterogeneity of all the CE risk factors decreased to varying degrees in the study design-specific subgroup analysis.

In general, the studies were widely geographically distributed, including seven Chinese provinces. The provinces of Ningxia, Qinghai, and Xinjiang were included in the study region-specific subgroup analysis, and had three risk factors, two risk factors, and one risk factor, respectively. Only one risk factor for AE was identified (participant sex in Ningxia, OR = 1.44, 95% CI: 1.11–1.86; *P* = 0.006). The significant risk factors for CE were participant sex in Ningxia (OR = 1.34, 95% CI: 1.03–1.75; *P* = 0.029) and Qinghai (OR = 1.49, 95% CI: 1.33–1.66; *P* < 0.001) and herdsman occupation in Xinjiang (OR = 1.73, 95% CI: 1.37–2.19; *P* < 0.001). The Supplementary Material shows this in more detail.

## Discussion

The pooled prevalence of AE and CE were 2.34% (95% CI: 1.74–3.13%) and 4.45% (95% CI: 2.53–7.71%), respectively, in the included individuals. However, the prevalence of AE was significantly higher than that previously calculated in China (0.96%) (3). Since we aim at understanding the risk factors for AE and CE, several studies on prevalence were not included due to restrictions in the search terms (i.e., “risk factor”), which could partially explain the discrepancy. Notably, a recent nationwide study revealed that the prevalence of CE in China has decreased to 0.07% (47), demonstrating that the current prevention and control measures have had a significant impact.

Sex, ethnicity, not washing hands before meals and herdsman occupation were found to be common risk factors for AE and CE in this meta-analysis. Similar to previous studies (48, 49), we found that women were more likely to develop echinococcosis than men, because they are more involved in housework, such as food preparation and pet care, and therefore have more opportunities to contact infected dogs, soil and vegetables (50). Furthermore, as a result of their increased number of regular abdominal ultrasound examinations to monitor reproductive health, women of childbearing age infected with echinococcosis have a greater chance of early detection (51), which will lead to detection bias. In a case-control study, Alaouadi (52) found that women are more susceptible to echinococcosis than men because women‘s higher estrogen levels might promote echinococcosis growth. Ethnicity could be a confounding factor because most Tibetans living in western China are herders (32), who thus regularly come into contact with infected canid definitive hosts.

The main route of human infection is through fecal-oral transmission because echinococcosis can spread *via* the ingestion of food, soil, or water contaminated with the feces of infected mammals (53). In line with previous study showing that no washing hand before meals was one of the risk factors for echinococcosis (27), and one meta-analysis on risk factors for global echinococcosis indicating that eating raw unwashed vegetables and drinking piped water were associated with higher odds of infecting through the accidental ingestion of worm eggs (54), the present analysis confirms the causal effects of poor hygienic habits on the higher risk of disease. Our study also revealed that nomadism is a risk factor for AE and CE, which is similar to the results of a previous meta-analysis conducted in Iran (19). Nomads live in areas with poor sanitation and economic disadvantages, where they are exposed to infected animals and have a higher risk of becoming infected.

The contribution of dogs to the spread of echinococcosis cannot be ignored. Dog ownership, feeding viscera to dogs, the presence of stray dogs and number of household dogs were all risk factors for CE, but there was insufficient evidence to conclude that these were also risk factors for AE. This finding contradicts previous studies, which reported that dog-related factors were linked to both AE and CE (55, 56). The high heterogeneity of dog-related factors for AE could partially help to explain the inconsistency; the potential mechanism however should be further studied. In China, there are a large number of pet dogs and stray dogs, and a previous meta-analysis found that the combined prevalence of *E. multilocularis* and *E. granulosus* in dogs was 7.3% (57). Furthermore, because dogs belonging to rural families are less likely to obtain nutritious food, their diets are supplemented by hunting small mammals, which are intermediate hosts of *E. multilocularis* (58), or by being fed livestock viscera, which supports the lifecycle of *E. granulosus* (59). The large number of infected dogs and close contact with dogs are the causes of the high rates of *E. multilocularis* and *E. granulosus* in humans.

Coming into contact with fox hides was not a significant risk factor in this meta-analysis. After sensitivity analysis, the I^2^ for this factor changed to 0.00, but overall effect was still not significant. However, previous studies have reported that exposure to foxes increases the risk of AE infection (50, 60). Thus, Schweiger (61) found an increase in the fox population starting in 1985; after 10–15 years, the number of human AE cases significantly increased. A plausible explanation could be urbanization (62), which has resulted in an increased number of foxes appearing in people‘s living quarters. Increased opportunities for people to come into contact with foxes has increased the infection risk in the human population.

**Figure 6:**
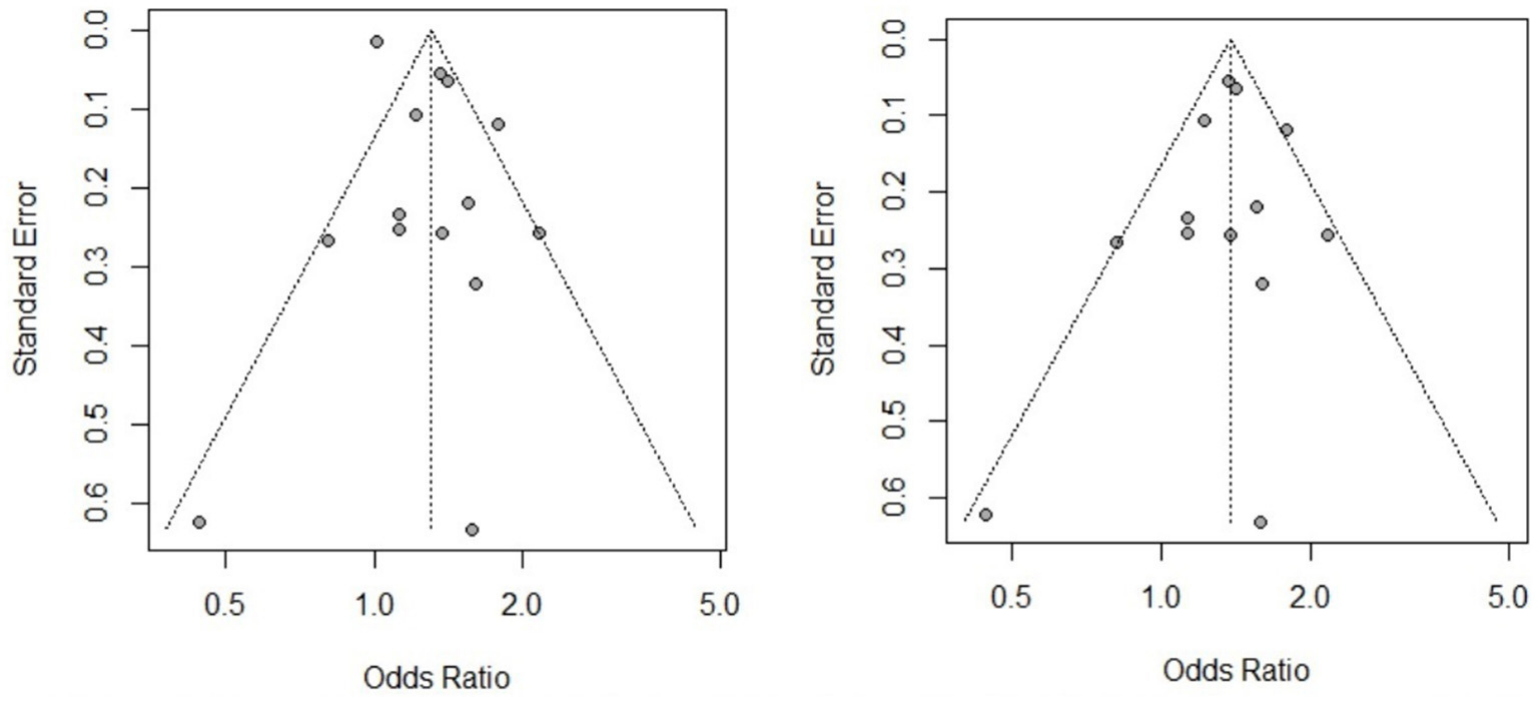
Funnel chart of CE risk factor sex before and after study design subgroup analysis.

The analysis of the overall effect of the CE-related risk factors sex, herdsman occupation and feeding viscera to dogs revealed publication bias. In our subgroup analysis based on study design, the publication bias disappeared, and the heterogeneity decreased. For example, as shown in Figure 6, when we excluded the case-control study, the CE risk factor sex became significant, and the funnel chart became symmetrical. Therefore, differences in study design may be the source of heterogeneity. We also conducted a subgroup meta-analysis based on study region. Sex was identified as a risk factor in several regions, but heterogeneity was not completely eliminated. As a result, it remains unclear whether the study region was the source of heterogeneity.

The results suggested that women and Tibetans should receive increased attention for echinococcosis prevention and control. Government-based interventions should be considered and implemented in these groups to raise awareness about the disease and take preventive measures. For instance, regular disease education and community screening should both be provided. Establishing good hygiene practices in the general population also prevents the long-term implications of echinococcosis. Dogs, the main vectors of transmission, play a key role in the prevention and control of echinococcosis. Thus, to improve public health, echinococcus infection in dogs must be properly managed and monitored, such as implementing monthly deworming and an effective registration of all dogs. In addition to the above suggestions, other echinococcosis control measures in China have been conducted, such as using EG95 antigen-based subunit vaccine to induce a robust immune response to infection in goats and sheep (63) as well as establishing the Belt and Road Network for the Elimination and Control of Echinococcosis and Cysticercosis (B&R-NEC), which provided the research and development capacity required to meet echinococcosis control targets (64). The establishment of an online scientific research platform and the use of animal vaccines can enable people to better understand and control the spread of echinococcosis.

Our study had several limitations, most relating to the lack of data available in the literature. Although we identified some risk factors for echinococcosis, more factors need to be analyzed, such as environmental and economic factors. Other limitations are related to the design of the included studies. All were observational studies (case-control and cross-sectional studies) that have inherent limits; for instance, observational studies are prone to selection bias and space bias (65). In addition, the timing of exposure and outcome could not be determined in these studies. Moreover, an insufficient number of regions were studies; specifically, only three areas were analyzed in the subgroup meta-analysis based on study region, and the details of echinococcosis risk factors in each region could not be identified.

## Conclusion

In summary, understanding the risk factors for echinococcosis provides a scientific basis to guide the formulation of prevention and control measures. Of the risk factors examined, for both AE and CE, the most important was ethnicity. Tibetans are at the highest risk of echinococcosis and thus must be closely monitored. The evidence for dog-related risk factors is also convincing, albeit at a lower level than that of ethnicity. Preventative measures of echinococcosis in humans should aim at raising the awareness of the disease in target groups and dog management. A series of national control measures, including regular dog deworming, public health education and community screening, should be implemented.

## Data Availability Statement

The original contributions presented in the study are included in the article/Supplementary Material, further inquiries can be directed to the corresponding author.

## Author Contributions

TZ and SL: study design and drafting of the manuscript. TZ, BL, and YL: data collection, analysis, and interpretation. All authors approval of the final version for publication.

## Funding

The study was supported by the National Natural Science Foundation of China (81860606) and the Natural Science Foundation of Qinghai Province (2019-ZJ-906).

## Conflict of Interest

The authors declare that the research was conducted in the absence of any commercial or financial relationships that could be construed as a potential conflict of interest.

## Publisher's Note

All claims expressed in this article are solely those of the authors and do not necessarily represent those of their affiliated organizations, or those of the publisher, the editors and the reviewers. Any product that may be evaluated in this article, or claim that may be made by its manufacturer, is not guaranteed or endorsed by the publisher.
