# Supplementary material for: Risk Factors Associated With Echinococcosis in the General Chinese Population: A Meta-Analysis and Systematic Review
Source: Front Public Health. 2022 May 17;10:821265. doi: 10.3389/fpubh.2022.821265 (PMC9152270; doi:10.3389/fpubh.2022.821265)
Supplement: Supplementary file 1 [file Data_Sheet_1.PDF]

Supplementary Materials

Table 1 The results of echinococcosis subgroup analysis divided by study design

Risk factor	Number of studies included	study design	Type of Echinococcosis	Sample size	positive cases	Test of heterogeneity			OR	95% CI	Test of overall effect	
						Q	P	I^2 (%)			Z	P-value
Dog ownership	4	case-control	CE	5793	456	3.52	0.319	14.7	1.37	1.33-1.83	2.19	0.029
Feeding viscera to dogs	3	case-control	CE	840	343	1.46	0.481	0	2.76	2.00-3.83	6.12	<0.001
Sex(Female/Male)	13	cross-sectional	CE	130879	3102	18.48	0.102	35.10	1.37	1.23-1.53	5.75	<0.001
Ethnicity(Tibetan/Han)	3	cross-sectional	CE	27965	1329	7.23	0.027	72.30	3.71	1.60-8.59	3.06	0.002
Drinking nonboiled water	3	cross-sectional	CE	14289	408	2.84	0.242	29.50	1.56	1.17-2.10	2.99	0.003
presence of stray dogs	3	cross-sectional	CE	11374	398	3.74	0.154	46.50	1.56	1.02-2.38	2.06	0.039
Herder status	6	cross-sectional	CE	154111	2900	33.3	<0.001	85.00	2.30	1.74-3.04	5.8	<0.001
Feeding viscera to dogs	4	cross-sectional	CE	13684	497	3.94	0.268	23.80	2.15	1.65-2.81	5.61	<0.001
Not washing hands before meals	4	cross-sectional	CE	14570	470	15.63	0.001	80.80	2.37	1.40-4.00	3.22	0.001

CE: cystic echinococcosis.

Table 2 The results of echinococcosis subgroup analysis divided by region

Risk factor	Number of sub-studies included	Type of Echinococcosis	Region (province)	Sample size	positive sample	Test of heterogeneity			OR	95%CI	Test of overall effect	
						Q	P	I^2(%)			Z	P-value
Sex (Female/Male)	3	AE	Ningxia	14008	257	1.69	0.429	0.00	1.44	1.11-1.86	2.73	0.006
Sex (Female/Male)	3	CE	Ningxia	14008	236	1.02	0.600	0.00	1.34	1.03-1.75	2.18	0.029
Drinking nonboiled water	3	CE	Ningxia	5463	305	22.78	<0.001	91.20	3.28	0.96-11.19	1.9	0.058
Sex (Female/Male)	4	CE	Qinghai	28565	1340	3.03	0.388	0.80	1.49	1.33-1.66	7.06	<0.001
Sex (Female/Male)	3	CE	Xinjiang	5092	90	9.97	0.007	79.90	1.02	0.43-2.42	0.05	0.964
Herder status	4	CE	Xinjiang	51616	379	4.86	0.182	38.30	1.73	1.37-2.19	4.57	<0.001

AE: alveolaris echinococcosis CE: cystic echinococcosis.

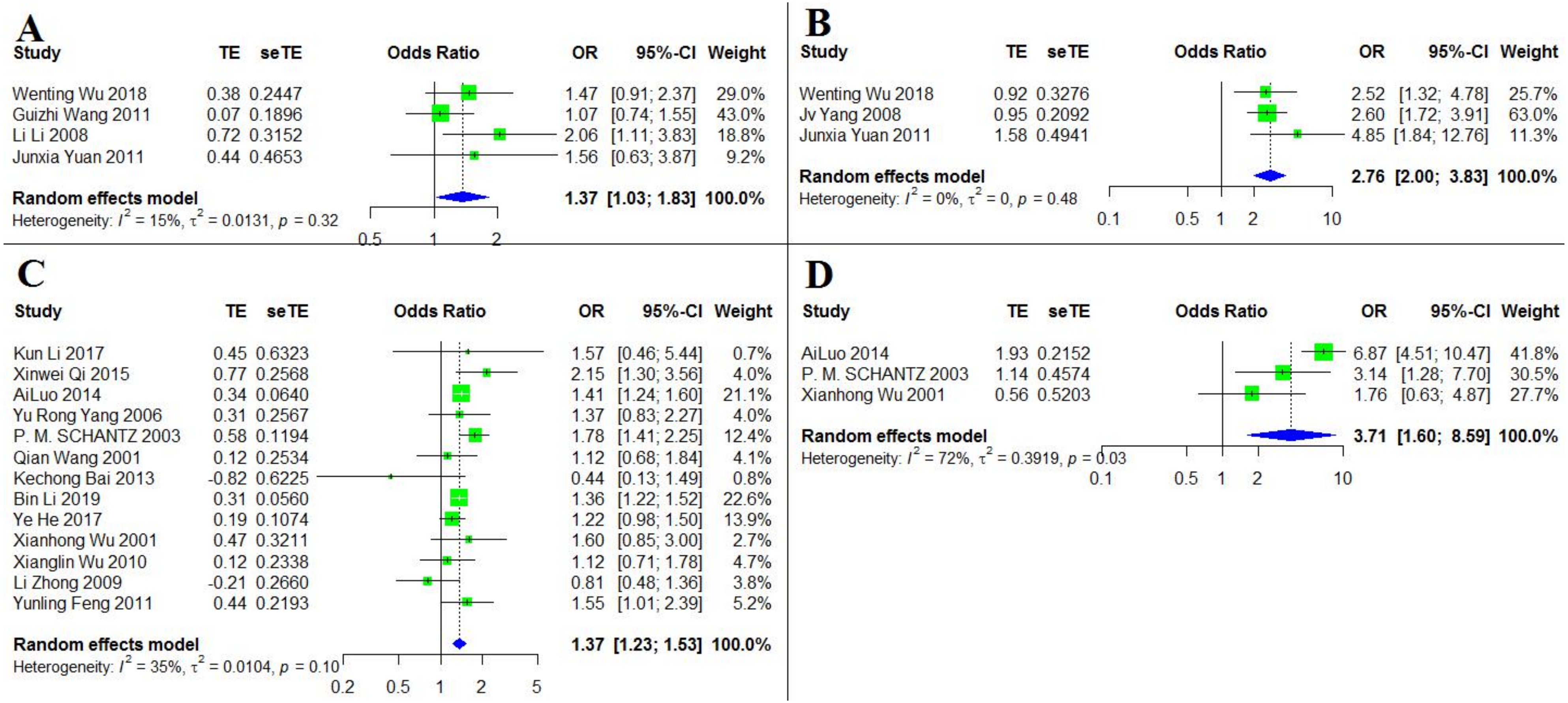

Figure 1 The forest chart of sub analysis for echinococcosis by study design.

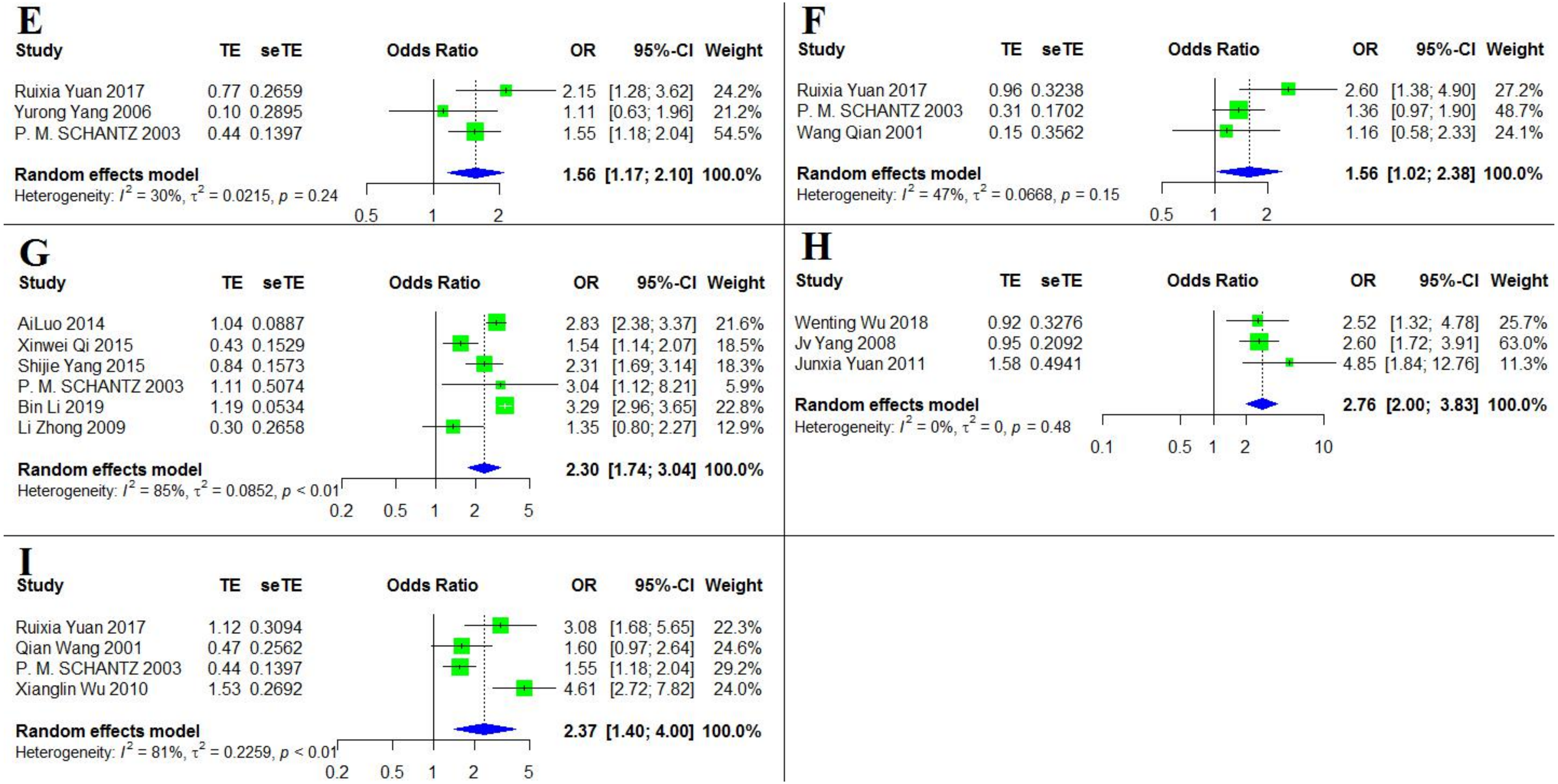

Figure 1 The forest chart of sub-analysis for echinococcosis by study design.(continue).

A: Dog ownership in case-control study; B: Feeding viscera to dogs in case-control study; C: Sex (Female/Male) in cross-sectional study; D: Ethnicity (Tibetan/Han) in cross-sectional study; E: Drinking nonboiled water in cross-sectional study; F: presence of stray dogs in cross-sectional study; G: Herder status in cross-sectional study; H: Feeding viscera to dogs in cross-sectional study; I: Not washing hands before meals in cross-sectional study.

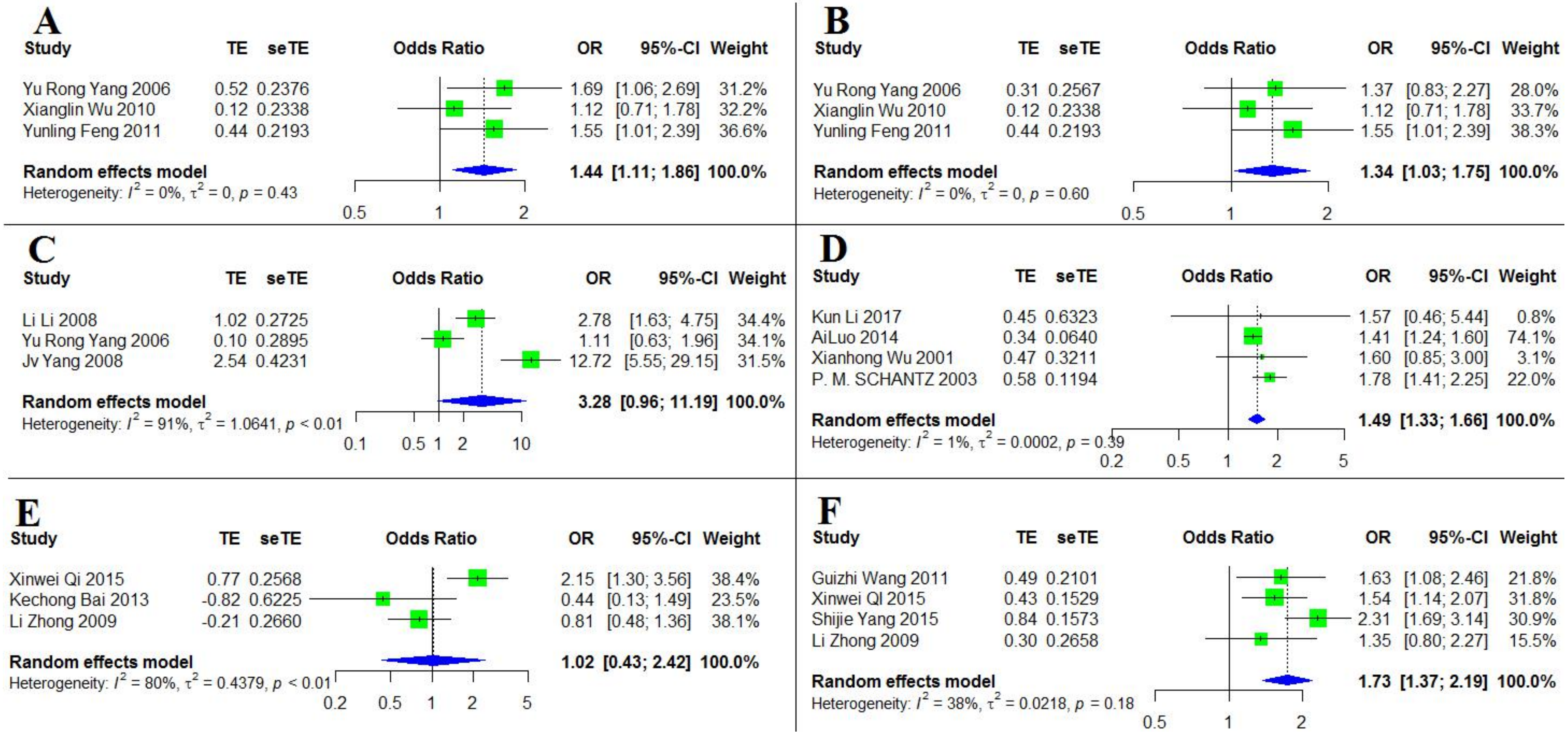

Figure 2 The forest chart of sub-analysis for echinococcosis by study region

A: Sex (Female/Male) for AE in Ningxia; B: Sex (Female/Male) for CE in Ningxia; C: Drinking nonboiled water for CE in Ningxia; D: Sex (Female/Male) for CE in Qinghai; E: Sex (Female/Male) for CE in Xinjiang; F: Herder status for CE in Xinjiang.

Table 3 The results of sensitivity analysis.

A. Sensitivity analysis for AE risk factor sex.

Omitting	OR	95% CI	p-value	τ^2	τ	I^2
P.M.SCHANTZ 2003	1.34	1.20-1.50	<0.001	0.000	0.008	0.2%
Patrick Giraudoux2013	1.50	1.25-1.80	<0.001	0.020	0.142	37.1%
Qian Wang 2001	1.45	1.24-1.69	<0.001	0.017	0.129	43.3%
Qian Wang 2006	1.41	1.27-1.60	<0.001	0.007	0.085	24.9%
Xianglin Wu 2010	1.48	1.27-1.72	<0.001	0.014	0.116	37.5%
Ye He 2017	1.51	1.30-1.76	<0.001	0.010	0.102	25.7%
YuRong Yang 2006	1.43	1.30-1.67	<0.001	0.015	0.122	39.9%
Yunling Feng 2011	1.44	1.23-1.69	<0.001	0.017	0.129	42.3%
Pooled estimate	1.45	1.26-1.66	<0.001	0.012	0.112	33.9%

B. Sensitivity analysis for AE risk factor ethnicity.

Omitting	OR	95% CI	p-value	τ^2	τ	I^2
P.M.SCHANTZ 2003	3.21	1.32-7.78	0.010	0.209	0.457	47.2%
Patrick Giraudoux2013	3.95	1.94-8.05	<0.001	0.000	0.000	0.0%
Qian Wang 2006	2.55	1.50-4.32	0.001	0.000	0.000	0.0%
Pooled estimate	2.93	1.81-4.75	<0.001	0.000	0.000	0.0%

C. Sensitivity analysis for AE risk factor dog ownership.

Omitting	OR	95% CI	p-value	τ^2	τ	I^2
P.S.Craig 2000	1.85	1.04-3.29	0.037	0.160	0.400	60.9%
Qian Wang 2001	1.55	0.88-2.74	0.133	0.186	0.431	75.5%
Qian Wang 2006	1.64	0.69-3.93	0.265	0.444	0.667	75.5%
Xianglin Wu 2010	1.31	1.01-1.70	0.043	0.014	0.119	17.5%
Pooled estimate	1.52	0.96-2.39	0.075	0.128	0.358	63.2%

D. Sensitivity analysis for AE risk factor playing with dogs.

Omitting	OR	95% CI	p-value	τ^2	τ	I^2
P.M.SCHANTZ 2003	3.25	1.73-6.12	<0.001	0.011	0.103	4.9%
Qian Wang 2001	1.57	0.24-10.12	0.64	1.720	1.312	95.1%
Qian Wang 2006	1.07	0.32-3.55	0.92	0.637	0.798	82.5%
Pooled estimate	1.72	0.45-6.52	0.42	1.247	1.117	91.8%

E. Sensitivity analysis for AE risk factor touch fox skin.

Omitting	OR	95% CI	p-value	τ^2	τ	I^2
P.S.Craig 2000	1.30	0.50-3.35	0.591	0.366	0.605	77.9%
Qian Wang 2001	1.44	0.72-2.90	0.303	0.164	0.405	64.1%
Qian Wang 2006	0.93	0.63-1.38	0.728	0.000	0.000	0.0%
Pooled estimate	1.19	0.70-2.02	0.523	0.129	0.359	58.5%

F. Sensitivity analysis for AE risk factor not washing hands before meals.

Omitting	OR	95% CI	p-value	τ^2	τ	I^2
P.M.SCHANTZ 2003	2.97	1.72-5.12	<0.001	0.148	0.384	63.8%
Qian Wang 2001	2.66	1.24-5.72	0.012	0.406	0.633	88.4%
Qian Wang 2006	2.22	1.07-4.62	0.033	0.358	0.598	86.4%
Xianglin Wu 2010	1.89	1.18-3.04	0.008	0.113	0.337	65.7%
Pooled estimate	2.40	1.34-4.28	0.003	0.283	0.532	82.8%

G. Sensitivity analysis for AE risk factor herder status.

Omitting	OR	95% CI	p-value	τ2	τ	I2
P.M.SCHANTZ 2003	2.71	2.13-3.45	<0.001	0.017	0.130	53.8%
Patrick Giraudoux 2013	3.14	2.38-4.15	<0.001	0.000	0.000	0.0%
Wang qian 2006	2.45	2.05-2.93	<0.001	0.000	0.000	0.0%
Pooled estimate	2.66	2.25-3.14	<0.001	0.003	0.050	8.0%

H. Sensitivity analysis for CE risk factor sex.

Omitting	OR	95% CI	p-value	τ^2	τ	I^2
AiLuo 2014	1.29	1.09-1.53	0.003	0.051	0.226	82.3%
Kechong Bai 2013	1.32	1.13-1.55	<0.001	0.050	0.223	86.3%
Bin Li 2019	1.29	1.09-1.54	0.004	0.057	0.240	82.2%
He Wei 2019	1.37	1.23-1.53	<0.001	0.010	0.102	35.1%
Kun Li 2017	1.30	1.11-1.52	0.001	0.051	0.226	86.5%
Li Zhong 2009	1.34	1.14-1.58	0.001	0.051	0.226	86.4%
P.M.SCHANTZ 2003	1.26	1.07-1.47	0.004	0.042	0.204	82.9%
Xinwei Qi2 015	1.26	1.08-1.48	0.004	0.047	0.216	85.3%
Qian Wang 2001	1.31	1.11-1.55	0.001	0.052	0.228	86.6%
Xianglin Wu 2010	1.32	1.11-1.55	0.001	0.052	0.228	86.6%
Xianhong Wu 2001	1.29	1.10-1.52	0.002	0.050	0.225	86.3%
Ye He 2017	1.31	1.10-1.56	0.002	0.055	0.235	86.3%
YuRong Yang 2006	1.30	1.10-1.53	0.002	0.051	0.226	86.4%
Yunling Feng 2011	1.29	1.09-1.51	0.003	0.050	0.224	86.1%
Pooled estimate	1.30	1.11-1.53	0.001	0.050	0.224	85.5%

I. Sensitivity analysis for CE risk factor ethnicity..

Omitting	OR	95% CI	p-value	τ^2	τ	I^2
AiLuo 2014	2.26	1.50-3.41	<0.001	0.000	0.000	0.0%
P.M.SCHANT Z2003	3.15	1.25-7.90	0.015	0.547	0.740	85.9%
Guizhi Wang 2011	3.71	1.60-8.59	0.002	0.392	0.626	72.3%
Xianhong Wu 2001	3.68	1.63-8.32	0.002	0.420	0.648	83.2%
Pooled estimate	3.18	1.55-6.52	0.002	0.403	0.635	79.2%

J. Sensitivity analysis for CE risk factor dog ownership.

Omitting	OR	95% CI	p-value	τ^2	τ	I^2
Junxia Yuan 2011	1.56	1.05-2.31	0.028	0.115	0.340	59.4%
Li Li 2008	1.46	0.99-2.15	0.055	0.096	0.309	51.8%
Qian Wang 2001	1.66	1.12-2.47	0.012	0.108	0.329	55.9%
Guizhi Wang 2011	1.73	1.19-2.52	0.004	0.063	0.252	35.5%
Wenting Wu 2018	1.59	1.02-2.50	0.042	0.150	0.387	59.5%
Xianglin Wu 2010	1.31	1.03-1.66	0.027	0.000	0.000	0.0%
Pooled estimate	1.54	1.09-2.17	0.014	0.087	0.295	49.4%

K. Sensitivity analysis for CE risk factor not washing hands before meals

Omitting	OR	95% CI	p-value	τ^2	τ	I^2
Xianglin Wu 2010	1.63	1.23-2.15	0.001	0.041	0.202	53.4%
Ruixia Yuan 2017	1.89	1.21-2.95	0.005	0.166	0.407	82.9%
P.M.SCHANTZ 2003	2.27	1.24-4.15	0.008	0.317	0.563	85.5%
Wenting Wu 2018	2.37	1.40-4.00	0.001	0.226	0.475	80.8%
Qian Wang 2001	2.20	1.32-3.66	0.003	0.223	0.472	86.0%
Pooled estimate	2.05	1.35-3.10	0.001	0.173	0.416	81.3%

L. Sensitivity analysis for CE risk factor herder status.

Omitting	OR	95% CI	p-value	τ^2	τ	I^2
Guizhi Wang 2011	2.30	1.74-3.04	<0.001	0.085	0.292	85.0%
AiLuo 2014	2.05	1.41-2.98	<0.001	0.171	0.413	87.6%
Xinwei Qi 2015	2.38	1.86-3.05	<0.001	0.060	0.246	78.7%
Shijie Yang 2015	2.16	1.58-2.94	<0.001	0.109	0.330	87.0%
P.M.SCHANTZ 2003	2.15	1.62-2.84	<0.001	0.098	0.314	87.6%
Bin Li2 019	1.99	1.49-2.65	<0.001	0.084	0.290	74.1%
Li Zhong 2009	2.34	1.79-3.05	<0.001	0.080	0.283	84.6%
Pooled estimate	2.19	1.67-2.86	<0.001	0.094	0.307	85.1%

M. Sensitivity analysis for CE risk factor feeding viscera to dog.

Omitting	OR	95% CI	p-value	τ^2	τ	I^2
DanLi2015	2.45	1.91-3.16	<0.001	0.031	0.176	32.8%
JunxiaYuan2011	2.19	1.83-2.64	<0.001	0.002	0.038	2.5%
JvYang2008	2.34	1.80-3.05	<0.001	0.031	0.175	28.9%
P.M.SCHANTZ2003	2.68	2.11-3.39	<0.001	0.000	0.000	0.0%
RuixiaYuan2017	2.24	1.79-2.80	<0.001	0.014	0.119	17.5%
WentingWu2018	2.38	1.85-3.05	<0.001	0.031	0.177	33.4%
XianglinWu2010	2.35	1.85-2.99	<0.001	0.028	0.166	31.5%
Pooledestimate	2.35	1.89-2.91	<0.001	0.018	0.134	21.5%

N. Sensitivity analysis for CE risk factor drink nonboiled water.

Omitting	OR	95% CI	p-value	τ^2	τ	I^2
Jv Yang 2008	1.77	1.26-2.49	0.001	0.065	0.255	54.8%
Li Li 2008	2.43	1.16-5.09	0.018	0.486	0.697	88.3%
P.M.SCHANTZ 2003	2.89	1.27-6.62	0.012	0.612	0.782	87.0%
Ruixia Yuan 2017	2.61	1.21-5.65	0.015	0.537	0.733	89.2%
YuRong Yang 2006	3.04	1.51-6.11	0.002	0.431	0.656	87.4%
Pooled estimate	2.47	1.36-4.47	0.003	0.377	0.614	85.7%

O. Sensitivity analysis for CE risk factor presence of stray dogs

Omitting	OR	95% CI	p-value	τ^2	τ	I^2
P.M.SCHANTZ 2003	2.04	1.17-3.56	0.012	0.115	0.338	47.3%
Ruixia Yuan 2017	1.54	1.00-2.38	0.052	0.065	0.256	42.2%
Qian Wang 2001	1.99	1.18-3.36	0.010	0.130	0.360	61.1%
Wenting Wu 2018	1.56	1.02-2.38	0.039	0.067	0.259	46.5%
Pooled estimate	1.75	1.15-2.65	0.008	0.090	0.300	50.9%

P. Sensitivity analysis for CE risk factor number of household dogs.

Omitting	OR	95% CI	p-value	τ^2	τ	I^2
Xiangman Zeng 2020	1.47	1.09-1.98	0.011	0.034	0.183	72.1%
Wei He 2019	1.84	0.78-4.35	0.165	0.322	0.567	81.8%
Wenting Wu 2018	2.08	1.21-3.53	0.008	0.099	0.314	58.2%
Pooled estimate	1.66	1.17-2.35	0.004	0.062	0.249	73.2%

Q. Sensitivity analysis for CE risk factor nomadism.

Omitting	OR	95% CI	p-value	τ^2	τ	I^2
Dan Li 2015	3.11	1.40-6.91	0.005	0.246	0.496	73.6%
Wei He 2019	2.12	1.45-3.11	<0.001	0.000	0.000	0.0%
Qian Wang 2001	3.16	1.43-6.96	0.004	0.230	0.479	70.3%
Pooled estimate	2.71	1.65-4.47	<0.001	0.109	0.330	55.8%

R. Sensitivity analysis for CE risk factor eating raw vegetables.

Omitting	OR	95% CI	p-value	τ^2	τ	I^2
Ruixia Yuan 2017	1.86	1.42-2.42	<0.001	0.000	0.000	0.0%
Li Li 2008	2.05	1.32-3.19	0.001	0.000	0.000	0.0%
Jv Yang 2008	1.81	1.42-2.30	<0.001	0.000	0.000	0.0%
Pooled estimate	1.86	1.47-2.35	<0.001	0.000	0.000	0.0%

Table 4 The result of sensitivity analysis for sub-analysis divided by study design

A. Sensitivity analysis for CE risk factor dog ownership by case-control study.

Omitting	OR	95% CI	p-value	τ^2	τ	I^2
Wenting Wu 2018	1.40	0.90-2.18	0.130	0.063	0.251	40.2%
Guizhi Wang 2011	1.65	1.16-2.34	0.005	0.000	0.000	0.0%
Li Li 2008	1.24	0.93-1.63	0.139	0.000	0.000	0.0%
Junxia Yuan 2011	1.39	0.97-1.99	0.073	0.042	0.206	41.4%
Pooled estimate	1.37	1.03-1.83	0.029	0.013	0.115	14.7%

B. Sensitivity analysis for CE risk factor feeding viscera to dogs by case-control study.

Omitting	OR	95% CI	p-value	τ^2	τ	I^2
Wenting Wu 2018	3.02	1.79-5.10	<0.001	0.050	0.224	25.9%
Jv Yang 2008	3.15	1.71-5.79	<0.001	0.039	0.198	18.3%
Junxia Yuan 2011	2.57	1.82-3.64	<0.001	0.000	0.000	0.0%
Pooled estimate	2.76	2.00-3.83	<0.001	0.000	0.000	0.0%

C. Sensitivity analysis for CE risk factor sex by cross-sectional study.

Omitting	OR	95% CI	p-value	τ^2	τ	I^2
Kun Li 2017	1.37	1.23-1.54	<0.001	0.012	0.110	40.3%
Xinwei Qi 2015	1.35	1.22-1.50	<0.001	0.007	0.086	28.8%
AiLuo 2014	1.36	1.18-1.56	<0.001	0.019	0.137	40.0%
YuRong Yang 2006	1.37	1.22-1.54	<0.001	0.012	0.111	40.5%
P.M.SCHANTZ 2003	1.33	1.21-1.47	<0.001	0.005	0.069	18.9%
Qian Wang 2001	1.39	1.24-1.55	<0.001	0.011	0.106	38.2%
Kechong Bai 2013	1.39	1.26-1.53	<0.001	0.007	0.081	27.1%
Bin L i2019	1.37	1.19-1.58	<0.001	0.020	0.141	40.1%
Ye He 2017	1.40	1.24-1.58	<0.001	0.011	0.106	34.9%
Xianhong Wu 2001	1.372	1.22-1.53	<0.001	0.012	0.109	39.8%
Xianglin Wu 2010	1.39	1.24-1.55	<0.001	0.011	0.106	37.8%
Li Zhong 2009	1.40	1.27-1.54	<0.001	0.006	0.074	23.3%
Yunling Feng 2011	1.36	1.21-1.53	<0.001	0.012	0.110	39.5%
Pooled estimate	1.37	1.23-1.53	<0.001	0.010	0.102	35.1%

D. Sensitivity analysis for CE risk factor ethnicity by cross-sectional study.

Omitting	OR	95% CI	p-value	τ^2	τ	I^2
AiLuo 2014	2.44	1.24-4.78	0.010	0.000	0.000	0.0%
P.M.SCHANTZ 2003	3.77	1.00-14.21	0.050	0.770	0.878	82.9%
Xianhong Wu 2001	5.15	2.46-10.80	<0.001	0.179	0.423	58.3%
Pooled estimate	3.7110	1.60-8.59	0.002	0.392	0.626	72.3%

E. Sensitivity analysis for CE risk factor drinking nonboiled water by cross-sectional study.

Omitting	OR	95% CI	p-value	τ^2	τ	I^2
RuixiaYuan2017	1.44	1.10-1.89	0.007	0.005	0.067	8.0%
YuRongYang2006	1.69	1.28-2.23	<0.001	0.008	0.090	15.1%
P.M.SCHANTZ2003	1.56	0.82-2.98	0.178	0.141	0.376	64.6%
Pooled estimate	1.56	1.17-2.10	0.003	0.022	0.147	29.5%

F. Sensitivity analysis for CE risk factor presence of stray dogs by cross-sectional study.

Omitting	OR	95% CI	p-value	τ^2	τ	I^2
RuixiaYuan2017	1.32	0.98-1.78	0.071	0.000	0.000	0.0%
P.M.SCHANTZ2003	1.76	0.80-3.88	0.162	0.211	0.459	64.5%
QianWang2001	1.77	0.95-3.31	0.072	0.143	0.378	68.1%
Pooled estimate	1.56	1.02-2.38	0.039	0.067	0.259	46.5%

G. Sensitivity analysis for CE risk factor herder status by cross-sectional study.

Omitting	OR	95% CI	p-value	τ^2	τ	I^2
AiLuo 2014	2.15	1.42-3.24	<0.001	0.171	0.413	88.0%
Bin Li 2019	2.07	1.50-2.85	<0.001	0.090	0.300	76.1%
Li Zhong 2009	2.50	1.90-3.27	<0.001	0.067	0.260	84.0%
P.M.SCHANTZ 2003	2.26	1.68-3.02	<0.001	0.090	0.300	88.0%
Shijie Yang 2015	2.29	1.65-3.17	<0.001	0.098	0.314	87.2%
Xinwei Qi 2015	2.60	2.06-3.28	<0.001	0.041	0.203	73.7%
Pooled estimate	2.30	1.74-3.04	<0.001	0.085	0.292	85.0%

H. Sensitivity analysis for CE risk factor feeding viscera to dogs by cross-sectional study.

Omitting	OR	95% CI	p-value	τ^2	τ	I^2
Ruixia Yuan 2017	1.90	1.51-2.39	<0.001	0.000	0.000	0.0%
Dan Li 2015	2.29	1.57-3.35	<0.001	0.056	0.237	48.9%
P.M.SCHANTZ 2003	2.58	1.83-3.64	<0.001	0.000	0.000	0.0%
Xianglin Wu 2010	2.11	1.54-2.89	<0.001	0.032	0.178	38.6%
Pooled estimate	2.15	1.65-2.81	<0.001	0.019	0.138	23.8%

I. Sensitivity analysis for CE risk factor not washing hands before meals by cross-sectional study.

Omitting	OR	95% CI	p-value	τ^2	τ	I^2
P.M.SCHANTZ 2003	2.82	1.49-5.34	0.002	0.242	0.492	75.9%
Qian Wang 2001	2.72	1.32-5.61	0.007	0.347	0.589	86.4%
Ruixia Yuan 2017	2.20	1.16-4.18	0.016	0.269	0.519	85.1%
Xianglin Wu 2010	1.84	1.27-2.67	0.001	0.057	0.238	52.0%
Pooled estimate	2.37	1.40-4.00	0.001	0.226	0.475	80.8%

Table 5 The result of sensitivity analysis for sub-analysis divided by region

A. Sensitivity analysis for AE risk factor sex in Ningxia.

Omitting	OR	95% CI	p-value	τ^2	τ	I^2
YuRong Yang 2006	1.33	0.97-1.83	0.073	0.001	0.026	1.3%
Xianglin Wu 2010	1.61	1.18-2.21	0.003	0.000	0.000	0.0%
Yunling Feng 2011	1.38	0.92-2.05	0.118	0.028	0.166	33.2%
Pooled estimate	1.44	1.11-1.86	0.006	0.000	0.000	0.0%

B. Sensitivity analysis for CE risk factor sex in Ningxia.

Omitting	OR	95% CI	p-value	τ^2	τ	I^2
YuRong Yang2006	1.33	0.97-1.83	0.073	0.001	0.026	1.3%
Xianglin Wu2010	1.47	1.06-2.04	0.020	0.000	0.000	0.0%
Yunling Feng2011	1.23	0.888-1.76	0.232	0.000	0.000	0.0%
Pooled estimate	1.34	1.03-1.75	0.029	0.000	0.000	0.0%

C. Sensitivity analysis for CE risk factor drink nonboiled water in Ningxia.

Omitting	OR	95% CI	p-value	τ^2	τ	I^2
Li Li 2008	3.68	0.34-40.20	0.28	2.842	1.686	95.6%
YuRong Yang 2006	5.75	1.30-25.45	0.02	1.029	1.014	89.0%
JvYang 2008	1.77	0.72-4.35	0.22	0.343	0.586	81.3%
Pooled estimate	3.28	0.96-11.19	0.06	1.064	1.032	91.2%

D. Sensitivity analysis for CE risk factor sex in Qinghai.

Omitting	OR	95% CI	p-value	τ^2	τ	I^2
Kun Li 2017	1.53	1.30-1.82	<0.001	0.008	0.090	33.7%
AiLuo 2014	1.75	1.41-2.17	<0.001	0.000	0.000	0.0%
Xianhong Wu 2001	1.53	1.28-1.82	<0.001	0.009	0.093	32.8%
P.M.SCHANTZ 2003	1.42	1.25-1.60	<0.001	0.000	0.000	0.0%
Pooled estimate	1.49	1.33-1.66	<0.001	0.000	0.014	0.8%

E. Sensitivity analysis for CE risk factor sex in Xinjiang.

Omitting	OR	95% CI	p-value	τ^2	τ	I^2
Xinwei Qi 2015	0.73	0.46-1.19	0.208	0.000	0.000	0.0%
Kechong Bai 2013	1.32	0.51-3.45	0.571	0.412	0.642	85.8%
Li Zhong 2009	1.08	0.23-5.03	0.926	1.032	1.016	82.0%
Pooled estimate	1.02	0.43-2.42	0.964	0.438	0.662	79.9%

F. Sensitivity analysis for CE risk factor herder status in Xinjiang.

Omitting	OR	95% CI	p-value	τ^2	τ	I^2
Guizhi Wang 2011	1.74	1.26-2.40	0.001	0.045	0.213	57.6%
Xinwei QI 2015	1.81	1.32-2.48	＜0.001	0.036	0.190	45.7%
Shijie Yang 2015	1.53	1.23-1.90	＜0.001	0.000	0.000	0.0%
Li Zhong 2009	1.81	1.39-2.36	<0.001	0.026	0.160	46.8%
Pooled estimate	1.73	1.37-2.19	<0.001	0.022	0.148	38.3%

Table 6 Results of publication bias.

A. Results of Egger`s test

Risk factor	Type of echinococcosis	Egger`s test	
		t	P
Sex(Female/Male)	AE	1.07	0.324
Ethnicity(Tibetan/Han)	AE	6.26	0.101
Dog ownership	AE	0.326	0.775
Playing with dogs	AE	2.50	0.242
Touch fox skin	AE	0.69	0.614
Not washing hands before meals	AE	1.46	0.283
Herder status	AE	0.40	0.757
Sex(Female/Male)	CE	2.44	0.031
Ethnicity(Tibetan/Han)	CE	-1.07	0.396
Dog ownership	CE	1.59	0.186
Not washing hands before meals	CE	2.24	0.111
Herder status	CE	-2.72	0.042
Feeding viscera to dogs	CE	2.87	0.035
Drinking nonboiled water	CE	1.55	0.220
presence of stray dogs	CE	1.08	0.392
Number of household dog (with each addition)	CE	0.94	0.521
Nomadism	CE	1.78	0.326
Eating raw vegetables	CE	2.37	0.254

B. Results of Egger`s test for sub-analysis divided by study design

Risk factor	study design	Type of Echinococcosis	Egger`s test	
			t	P
Dog ownership	case-control	CE	1.44	0.287
Feeding viscera to dogs	case-control	CE	1.35	0.406
Sex(Female/Male)	cross-sectional	CE	-0.59	0.568
Ethnicity(Tibetan/Han)	cross-sectional	CE	-5.46	0.115
Drinking nonboiled water	cross-sectional	CE	-0.05	0.970
presence of stray dogs	cross-sectional	CE	0.43	0.744
Herder status	cross-sectional	CE	-2.12	0.101
Feeding viscera to dogs	cross-sectional	CE	1.53	0.265
Not washing hands before meals	cross-sectional	CE	1.51	0.270

C. Results of Egger`s test for sub-analysis divided by study region.

Risk factor	Region (province)	Type of Echinococcosis	Egger`s test	
			t	P
Sex(Female/Male)	Ningxia	AE	-0.16	0.898
Sex(Female/Male)	Ningxia	CE	-0.35	0.787
Drinking nonboiled water	Ningxia	CE	1.33	0.411
Sex(Female/Male)	Qinghai	CE	0.71	0.549
Sex(Female/Male)	Xinjiang	CE	-0.71	0.606
Herder status	Xinjiang	CE	-0.82	0.500

Table 7 Abbreviation table

Full name	Abbreviation
Chinese National Knowledge Infrastructure	CNKI
Chongqing VIP Information	VIP
Odds ratio	OR
Confidence interval	CI
Cystic echinococcosis	CE
Alveolar echinococcosis	AE
Echinococcus granulosus	E. granulosus
Echinococcus multilocularis	E. multilocularis
Disability-adjusted life years	DALYs
Newcastle-Ottawa Scale	NOS
Agency for Healthcare Research and Quality	AHRQ
